# Cardiac Manifestations of Rheumatological Conditions: A Narrative Review

**DOI:** 10.5402/2012/463620

**Published:** 2012-10-17

**Authors:** Mohammad Bagher Owlia, Seyed Mohammad Yousof Mostafavi Pour Manshadi, Nafiseh Naderi

**Affiliations:** ^1^Department of Medicine, Shahid Sadoughi Hospital, Shahid Sadoughi University of Medical Sciences, Yazd, Iran; ^2^Department of Medicine, Ali ben Abitaleb Medical College, Islamic Azad University, Yazd, Iran

## Abstract

Cardiovascular diseases are common in systemic rheumatologic diseases. They can be presented at the time of diagnosis or after diagnosis. The cardiac involvements can be the first presentation of rheumatologic conditions. It means that a patient with rheumatologic disease may go to a cardiologist when attacked by this disease at first. These manifestations are very different and involve different structures of the heart, and they can cause mortality and morbidity of patients with rheumatologic diseases. Cardiac involvements in these patients vary from subclinical to severe manifestations. They may need aggressive immunosuppressive therapy. The diagnosis of these conditions is very important for choosing the best treatment. Premature atherosclerosis and ischemic heart disease are increased in rheumatoid arthritis and systemic lupus erythematosus, and may be causes of mortality among them. The aggressive control of systemic inflammation in these diseases can reduce the risk of cardiovascular disease especially ischemic heart disease. Although aggressive treatment of primary rheumatologic diseases can decrease mortality rate and improve them, at this time, there are no specific guidelines and recommendations, to include aggressive control and prevention of traditional risk factors, for them.

## 1. Introduction

Systemic rheumatic diseases are autoimmune inflammatory conditions that involve several organs, frequently involving the blood vessels and the heart. Cardiac disease may occur in patients with a definite diagnosis of a rheumatologic disorder, or may be the initial manifestation in patients with no prior diagnosis. Cardiac involvements in rheumatic diseases can show themselves in different ways from asymptomatic or mild to severe or life-threatening and are significant causes of morbidity and mortality in patients with rheumatic disorders. Rheumatologic diseases can be considered as causes of myocardial, valvular, and pericardial and conduction system abnormalities. Because of these abnormalities, it is thought that rheumatologic disorders have been associated with premature atherosclerosis leading to ischemic heart disease at young ages. The increased risk of coronary disorders cannot be solely attributed to traditional cardiovascular (CV) risk factors, and may be a result of chronic systemic inflammation from the rheumatic disease. The prevalence and importance of cardiovascular disease in rheumatologic disorders have increased in the setting of therapeutic advances. One should consider chronic inflammation as a cause of cardiac diseases in people with and without chronic inflammatory joint disease. Treatments to suppress inflammation have potential benefit in reducing cardiovascular disease morbidity and improving musculoskeletal function. Cardiovascular morbidity and mortality rate is higher in association with many of the rheumatic conditions than normal conditions. In particular, coronary heart disease seems to be associated with inflammatory rheumatic conditions. It is likely that chronic systemic inflammation increases accelerated atherosclerosis in these patients. While classic and enthusiastic involvement of heart is devoted to acute rheumatic fever (ARF) [1], specific rheumatic diseases are commonly associated with heart involvement [2–7]. 

## 2. Rheumatoid Arthritis 

Rheumatoid arthritis (RA) is a common chronic autoimmune disease. It is more common in women than in men (2 to 4 times) [3]. It is seen in people in the third and fourth decades of their life. Its prevalence is about 1% as reported by Roman and Salmon [3], and up to 50% of patients have asymptomatic cardiac involvement [2]. The diagnosis of RA is based on clinical manifestations. Cardiac involvements are common in RA patients and there are two groups of lesions in this organ: (i) rheumatoid granulomata, and (ii) non-specific inflammatory lesions. More recent studies have shown that these lesions are manifestations of the rheumatoid. Cardiovascular disorders can lead to death in RA patients. They are responsible for about half the death of patients with RA [8]. It is an unknown cause of higher rates of coronary disease in rheumatoid patients. The most mortality associated with RA is due to cardiovascular disease, especially because of ischemic heart disease [6, 9, 10]. A minor study similarly detected a relation between carotid atherosclerosis and RA duration as well as more extensive extraarticular disease [11]. One of the cardiac manifestations of RA is premature atherosclerosis, especially in the carotid. The prevalence of carotid atherosclerosis in RA is high [3, 11]. Cardiac involvements in RA include pericarditis, valvulitis, myocarditis, and an increased prevalence of atherosclerotic coronary heart disease. The pericardium is affected in approximately 40% of patients, with pericarditis being the most frequent cardiac manifestation in RA [2, 4]. Pericarditis is more common in patients with rheumatoid nodules and a positive RF [2]. Silent pericardial effusion is seen more frequently than acute symptomatic pericarditis in patients with RA [12]. Constrictive pericarditis is not common but can occur [13]. Myocardial disease in RA is rare and does not typically have clinical symptoms [2]. The risk of congestive heart failure is high in RA patients. Heart failure may be one of the main causes of increased cardiovascular mortality in RA, particularly in men [14–16]. Diastolic LV dysfunction on Echo-Doppler was found more in RA patients than in the general population [17]. Secondary amyloidosis in the past was found in the rheumatoid hearts but is now rare in rheumatoid disease, and can cause cardiomyopathy and AV block [2], though conduction abnormalities have been reported [5, 18]. Echocardiographic and autopsy studies show evidence of valvular disease in almost 30% of patients with RA [19]. As compared to normal population, mitral regurgitation may be more common in RA patients. Aortic root abnormalities, including aortitis, have been reported in association with RA, but are still rare [2, 19]. Coronary vasculitis is a rare complication of RA, but patients with RA have an increased risk of CAD and premature death from atherosclerotic disease [2]. In one of the studies, valvular disease was significantly more common in the RA group than in the control group (83 versus 53%, *P* = 0.02). The most common valvular abnormality was mitral regurgitation (MR), which was found in 24 patients (80%). Mitral valve prolapse was demonstrated in 5 patients. Aortic valve disease was not significantly more common in the RA patients than in the controls. Ten of the cases (33%) had aortic regurgitation (AR), and one had aortic stenosis [5]. Villecco et al. described right bundle branch block in 35% of 60 patients with RA [18, 20]. It was discovered that AV block is rare in RA, but usually complete. Ahern et al. described congenital complete heart block (CHB) in 0.1% of the patients with RA, especially in females, and concluded that it is more common in patients with subcutaneous nodules [21]. According to one study, RA is associated with an increased risk of cardiovascular and/or cerebrovascular disease morbidity due to MI, CHF, and probably CVA, and may be an independent risk factor for these events [22].

Overall, the cardiac manifestations of RA include pericarditis, cardiomyopathy/myocarditis, cardiac amyloidosis, coronary vasculitis, arrhythmia, and valve diseases; congestive heart failure and ischemic heart diseases are more common and associated with an increased mortality rate in these patients when compared with the general population [7]. 

A small part of RA patients who had a long duration of the disease had asymptomatic valvular lesions [23]. Aortitis and aortic insufficiency may occur as an unusual complication of rheumatoid arthritis [24–27]. Other studies showed that in patients with rheumatoid arthritis (RA), a major cause of sudden cardiac death is atherosclerotic coronary artery disease, leading to acute coronary syndrome and ventricular arrhythmias [4, 18]. In a case report, aortic valve aneurysm was reported in an RA patient [28], and in another case report, aortic insufficiency was reported in a patient with RA [29]. The results of another study showed that RA is an important etiological factor for cardiac involvement [30]. Pericarditis may be the most common cardiac manifestation in RA patients, and the incidence in autopsy cases is more than 30% [31]. In the results of a study carried out on cardiac manifestations, cardiac manifestations were observed in 40 (41%) patients with RA. Pericarditis appeared in 11 patients, valvulopathy in 12 patients, and coronaropathy in 11 patients [32]. In another study that was examined, computed tomography was observed in 13.6% of the cases, with an effusion of the pericardium in rheumatoid arthritis [33]. An echocardiographic study of 44 patients with active rheumatoid arthritis was carried out by means of a Picker ultrasonic laminograph. A posterior pericardial effusion was found in 14 (32%) patients, and pericardial thickening in 5 (11%) patients. The maximum amplitude of the anterior cusp of the mitral valve was reduced in 18 patients, and the diastolic (EF) slope was abnormal in 17 patients. The overall incidence of cardiac involvement in these patients was 73% [34]. In a retrospective study of 172 patients with juvenile rheumatoid arthritis, symptomatic cardiac involvement occurred in 13 (7.6%) patients [35]. Pericarditis occurred in seven patients, perimyocarditis in four, and myocarditis in two patients. One of the patients had myocarditis associated with cardiac tamponade. Among the 172 patients with juvenile rheumatoid arthritis, five children died, while four belonged to the symptomatic cardiac involvement group (*P* < 0.001). Pericarditis is known to occur in rheumatoid arthritis and is not rheumatic in origin. In another study, pericarditis was diagnosed clinically in 20 of a series of 285 cases of juvenile rheumatoid arthritis (7%). Out of 11 postmortem studies carried out on patients with juvenile rheumatoid arthritis, pericarditis was found in 5 (45%). In a patient with juvenile rheumatoid arthritis, pericarditis may occur at any age [36].

## 3. Systemic Lupus Erythematosus (SLE)

Systemic lupus erythematosus (SLE) is a chronic systemic autoimmune disease whose prevalence is almost 1 : 2500 as reported by Roman and Salmon and Lawrence et al. [3, 37], and almost 15 to 50/100000 people as reported in the United States. In SLE, involvement of women is more than men, in that almost 90% of patients are women [2]. Black men and women are more involved than whites and the manifestations of the disease are more severe in them than in whites [37]. We can diagnose SLE with systemic clinical and laboratory manifestations [3, 38]. Cardiac involvement is a common and significant cause of morbidity and mortality in SLE patients. Its prevalence is more than 50% [39]. The pathogenesis of SLE is unknown but may be because of the multi factors presented such as genetic, environmental, hormonal, and immunologic factors. The cardiovascular manifestations of SLE are valvular heart diseases associated with Libman-Sacks lesions, serositis associated with pericardial disease, and venous and arterial thrombosis associated with antiphospholipid antibodies [3]. The factors related to SLE that is associated with clinical manifestations of artery coronary disease are older age at diagnosis [3, 40], longer duration of SLE [3, 40, 41], longer duration of treatment with corticosteroids [3, 42, 43], higher damage score [41], higher levels of homocysteine, and low density lipoprotein cholesterol [43].The case-control studies indicate that premature atherosclerosis is a significant result of SLE itself [44–47]. In SLE, arterial stiffness is increased even without atherosclerosis; it is related to the duration of the disease, C-reactive protein levels, and interleukin-6 [48]. Abnormalities of structure and function of LV have been seen in SLE patients [49, 50]. Also, we observed higher LV mass index in patients with SLE, as a result of the Starling phenomenon (higher end diastolic and comparable end systolic dimensions) [51]. Myocarditis is a rare manifestation of SLE diagnosed clinically or detected at autopsy associated with the activity of the disease [52, 53]. Even though valvular nodules have been observed in patients with SLE, the clinical manifestation of valvular heart disease is much less common in SLE [3]. Echocardiographic studies showed different frequencies of vegetations or nodules detected on the mitral (7 to 15%) and aortic (3 to 19%) valves [3, 47]. Significant valvular heart disease included <20% of patients undergoing Doppler echocardiography [47, 54]. The progression of severe valvular regurgitation may be related to high levels of IgG anticardiolipin antibodies [54]. Large transthoracic echocardiographic studies show an association between high levels of anticardiolipin antibodies, valvular nodules, and regurgitation, especially those involving mitral valve [47, 55, 56]. Pericardial disease is the most common clinical cardiovascular manifestation of SLE. However, 20 to 50% of SLE patients in relatively large series had clinical manifestation of pericarditis with or without pericardial effusion [50, 55, 57, 58]. Pericardial effusion most commonly occur in the severe level of the activity of the disease (flares), but may be asymptomatic [50]. Moderate to large pericardial effusions were reported in 7% of patients in one series [50]. Cardiac tamponade is rare without renal failure. The other rare cardiac manifestation of SLE is pericarditis constrictive [2]. Myocarditis is uncommon in SLE that includes autopsy studies. Myocardial abnormalities are more common in echocardiography than clinical manifestations [59]. Endocarditis is another cardiac manifestation of SLE that is more common in echocardiography studies than clinical manifestations [2]. Arrhythmias can occur in patients with SLE. The most common arrhythmia is sinus tachycardia. It can be seen in active disease, and can be resolved with treatment of SLE [2]. The infants from mothers with anti-Ro or anti-La without diagnosis of SLE have an increased incidence of congenital complete atrioventricular (AV) block [60]. Coronary arthritis is another rare cardiac manifestation whose diagnosis is difficult [61]. Aortitis can occur rarely in SLE [62]. Pulmonary artery hypertension is common but is usually mild. It may be without clinical manifestation which is diagnosed initially by echocardiography [63]. In a study conducted on echocardiography, variable valve diseases such as mitral valve thickening or vegetation, mitral valve prolapsed, and aortic valve vegetation; mitral, aortic, and tricuspid regurgitation; mitral stenosis are reported. The conclusion of this study was that valvular heart involvement is common in patients with SLE [64]. In another investigation, mitral annulus calcification and aortic valve calcification are common in young patients with SLE [65]. Mitral prolapse is more prevalent in SLE patients [66].

## 4. Systemic Sclerosis 

Systemic sclerosis (SSc) is a systemic autoimmune disease that is diagnosed with fibrosis of the variable tissues because of the gathering of collagen and other extracellular proteins. Its etiology is unknown but its pathophysiology involves microvascular abnormalities, secondary ischemia, and fibroblast overactivity. Its prevalence is 2 in 10000 and is commonly seen in women [37]. Cardiac manifestations in SSc are different from silent involvement to overt clinical signs associated with increasing mortality and morbidity [67]. One of the cardiac manifestations of systemic sclerosis is myocardial abnormalities, including segmental wall motion abnormalities, and impaired coronary flow reserve in the absence of epicardial coronary artery disease, and coronary vascular diseases [68, 69]. True myocardial abnormality is more common in SSc patients with diffuse disease and peripheral skeletal myositis. Abnormalities of right and/or left ventricular are observed for both of them [70]. Microvascular abnormalities are base events in patients with systemic sclerosis. Recent studies also show the involvement of large arteries in patients. The stiffness of the microvascular and large arteries has been reported [71]. A large investigation of 106 patients with systemic sclerosis showed decreased aortic distensibility in comparison to the control group [72]. In patients with systemic sclerosis, diffuse conduction abnormalities and arrhythmias are seen as detected by electrocardiography [73]. The most common arrhythmia in SSc patients is premature ventricular contraction. The risk of CHF and cardiac sudden death is increased in SSc patients with coexistent skeletal myositis. Primary valvular disease is uncommon in patients with systemic sclerosis [67]. Pulmonary artery hypertension is a serious clinical manifestation in SSc patients [74]. Pericardial disease is detected in autopsy studies in patients with systemic sclerosis [75]. Clinical manifestations of systemic sclerosis are very rare [3]. In large echocardiography studies, small pericardial effusion was reported in 14% of 77 patients with SSc [72].

## 5. Ankylosing Spondylitis

Ankylosing spondylitis (AS) is a systemic inflammatory disease that involves the whole spine and sacroiliac joints. It may have some effects on peripheral joints. The main characteristic of musculoskeletal lesion is inflammation of enthesis (the site where tendons and ligaments attach to bones). The prevalence of ankylosing spondylitis is about 1 to 2 per 1000 among whites, and its ratio for males to females is 3 : 1 to 4 : 1 [3, 37]. One of the cardiac manifestations of ankylosing spondylitis is aortic disease, which includes aortic regurgitation and/or aortitis that is recognized even before ankylosing spondylitis was diagnosed. Mitral regurgitation was also reported in AS patients. The other aortic disease is thickening of aortic wall. Mild aortic root dilatation has been reported in patients with ankylosing spondylitis [3, 76]. Another cardiac manifestation of ankylosing spondylitis is conduction abnormalities from 2 to 20% among patients [76–78]. The most common conduction abnormality in patients with ankylosing spondylitis is first-degree atrioventricular block. Higher grade atrioventricular block and right and left bundle-branch block have also been seen in ankylosing spondylitis patients [3, 76]. Atrial fibrillation was reported in AS, especially in patients with HLA-27 [78]. Myocardial dysfunction including diastolic filling abnormalities is another cardiac manifestation of ankylosing spondylitis [3, 76, 77]. Myocardium and pericardium can rarely be affected, though pericarditis is rare in AS [2]. Peripheral vascular disease and congestive heart failure are more common in ankylosing spondylitis patients than in the general population; this causes higher death rate in these patients than in the general population [3, 79]. Another cardiac manifestation in AS that is detected in electrocardiography is QT dispersion (QTd) which is greater in ankylosing spondylitis patients than in the healthy population [76].

## 6. Antiphospholipid Syndrome

Antiphospholipid syndrome (APS) is an autoimmune systemic disease that is associated with thrombotic arteries and veins and recurrent fetal loss [80]. APS is the cause of different cardiac abnormalities. The most common cardiac involvement in APS is valvular disease [81, 82] with prevalence of 82% detected by transesophageal echocardiography [81], including verrucous endocarditis which leads to valvular thickening, insufficiency [80] and vegetation [82]. APS can also cause coronary artery bypass graft occlusion and premature myocardial infarction [80]. APS causes accelerated atherosclerosis which leads to cardiac disease. Cardiovascular mortality is increased in patients with APS. Other cardiac manifestations of APS include ventricular dysfunction, intracardiac thrombi, myxomas, endocardial disease, myocardial involvement, microvascular thrombosis, and pulmonary hypertension [81–83]. A major clinical cardiac manifestation of APS is arterial thrombosis [84]. In one study conducted on valvular involvements, the prevalence of valvular disease in patients with APS was 19%; the most common valvular involvement was mitral (91%), and the most common lesion was mitral insufficiency [85].

## 7. Psoriatic Arthritis 

Psoriatic arthritis is a combination of inflammatory arthritis and psoriasis diseases. This disease affects 2 to 11% of those who have psoriasis [86, 87]. Studies in Norway and United States show prevalence of 1 to 2 per 1000, in which men and women are equally affected [86, 88]. Its cause is unknown, and may be modulated by immunologic, genetic, and environmental factors. A recent echocardiographic study from Spain showed that 50 patients with psoriatic arthritis without cardiovascular risk factors had valvular regurgitation, normal pulmonary artery pressures, and abnormal diastolic relaxation as compared to 50 patients of the control group [89]. A recent US study showed that psoriatic arthritis patients had higher prevalence of cardiovascular disease risk factors and ischemic heart disease, peripheral vascular disease, and congestive heart failure as compared to the control group [79]. In a large UK study of 130976 patients with psoriasis, especially in younger patients having severe disease, the risk of having myocardial infarction increased [90]. 

## 8. Polymyositis and Dermatomyositis

Polymyositis and dermatomyositis are chronic systemic inflammatory diseases that are diagnosed by muscles' weakness and fatigue, and histopathologically by inflammatory cells that infiltrate the skeletal muscle. Extra organs' involvements are common in poly- and dermatomyositis. One of them is cardiac involvement [91]. One recent long-term followup study showed that the poly- and dermatomyositis patients with cardiac involvement had poor prognosis, and cardiac involvements like heart failure, arrhythmia, cardiac arrest and myocardial infarction are the most common cause of death in myositis patients [92]. Cardiac involvement was reported in 10 (20%) patients with polymyositis [91–93]. In another cohort study, it was reported that cardiac involvement is a cause of death in polymyositis (myositis) patients [92]. The cardiac manifestations in poly- and dermatomyositis are common. The most common of these manifestations is congestive heart failure. It is reported in about 45% patients with polymyositis [91, 94]. The other cardiac manifestation in myositis is left ventricular diastolic dysfunction that is reported in 42% of myositis patients. Coronary artery disease and myocardial infarction were also reported. Pericarditis is uncommon in patients with myositis [91]. ECG abnormalities are more common in polymyositis and dermatomyositis. These abnormalities include atrial and ventricular arrhythmias, bundle branch block, A-V blocks, high-grade heart block, prolongation of PR-intervals, ventricular premature beats, left atrial abnormality, abnormal Q-waves, and nonspecific ST-T wave changes. In one study, conduction abnormalities were reported as the most common cardiac manifestations of polymyositis and dermatomyositis. The most conduction abnormalities were bundle branch block and A-V block [91, 95]. The major cardiac involvement in PM/DM is myocarditis with variable degree of fibrosis and small vessel disease [96]. 

## 9. Vasculitides

The primary systemic vasculitides are inflammatory diseases whose cause is unknown. They are classified by size of vessels and the pattern of organs involvement. The vasculitides that are more associated with cardiovascular diseases (CVD) include giant cell arteritis (GCA), Takayasu arteritis (TA), polyarteritis nodosa (PAN), and Churg-Strauss syndrome (CSS) [2]. Cardiac involvement is not usual in Wegener's granulomatosis and we do not discuss it in this paper. Conduction abnormalities and accelerated atherosclerosis may occur in patients with Wegener's granulomatosis [97, 98].

## 10. Takayasu Arteritis (TA)

Takayasu arteritis (TA) is an idiopathic disease that involves the large vessels. It is rare, and its prevalence is 2.6/1000000 in the United States and 1.26/1000000 in northern Europe. It is more common in Japan. Its prevalence has been reported as 1 in every 3000 autopsies [99]. It is more common in young women than men [3]. Cardiac manifestations result from aortic involvement. This involvement is aortic aneurysm which leads to aortic regurgitation and inadequately treated hypertension. Coronary artery vasculitis is not common [100, 101]. Left ventricular systolic dysfunction is another cardiac manifestation of TA that is seen in about 18% of patients with TA [102]. TA can lead to vessels wall thickening, fibrosis, and stenosis. In unusual cases, it can mimic infective endocarditis [103]. In one case report, coronary arteritis was reported as an unusual cardiac manifestation of TA [104]. In other studies, aortic valve regurgitation and heart failure were reported as cardiac involvements in TA patients [105, 106]. Aortic valve regurgitation can occur alone as a sole cardiac involvement of TA patients [107].

## 11. Giant Cell Arteritis (GCA)

Giant cell arteritis (GCA) is more common in the older population whose age is more than 50 years. The mean age of patients is 74 years. GCA involves the large vessels. Its cardiac involvement is aortitis that can affect the primary branches of aorta. The other cardiac manifestations in GCA patients are thoracic aortic aneurysms which are 17 times more common than patients without GCA and abdominal aortic aneurysms that are 2.5 times more than patients without GCA. Another manifestation of GCA is thoracic aortic dissection which leads to increased mortality in GCA patients [108]. Coronary artery disease, aortic valve insufficiency, and left ventricular dysfunction are other cardiac involvements [109, 110]. Myocardial infarction is rare in patients with giant cell arteritis [111]. In one case report, cardiogenic shock was reported as one of the manifestations of GCA [110].

## 12. Polyarteritis Nodosa (PAN)

Polyarteritis nodosa (PAN) is a disease that affects medium-size arteries [2, 112]. It is rare, and its prevalence is less than 1/100000 in a year. The predominance of men and women is the same. It can occur in different ranges of age, but its incidence peak is between 40 and 60 years. PAN has cardiac manifestations, the most common of which is hypertension. Other cardiac manifestations include angina, myocardial infarct, congestive heart failure, and left ventricular hypertrophy, though coronary arteritis may also occur [2]. In one case report, aortic dissection was reported as one of the cardiac manifestations of PAN [113]. Pericarditis is rare in patients with PAN [2].

## 13. Churg-Strauss Syndrome (CSS)

Churg-Strauss syndrome (CSS) is a rare disease that involves small vessels. Its prevalence is 2.4/1000000 annually. It involves all ages and genders. There is no predominance between male and female, and its age peak is 35 to 50 years old. The frequency of cardiac involvements in CSS is different, that is, from 15 to 55%. Cardiac involvement is a major cause of death in patients with CSS. It is a cause of mortality in half of CSS patients. Most common cardiac manifestations of CSS patients are pericarditis, myocarditis, and less common, coronary arthritis. Congestive heart failure may occur in 15 to 30% of patients with CSS. Myocardial and epicardial granuloma can be seen. In one case report, acute myocarditis and cardiogenic shock were reported as cardiac manifestations of Churg-Strauss syndrome [114]. In another case report, myopericardial involvement was reported as a cardiac manifestation of Churg-Strauss syndrome [114]. In one study, supraventricular and ventricular arrhythmias were reported as cardiac manifestations of CSS which occurs frequently [115]. Left ventricular systolic dysfunction was also reported as a cardiac manifestation in CSS [116, 117]. In one study, pericardial effusion (41%) and mild to severe valvular insufficiency (73%) were reported as cardiac manifestations of patients with CSS [117]. In one case report, acute coronary syndrome was reported as a cardiac manifestation of CSS [118]. 

## 14. Behcet's Disease (BD)

Cardiac manifestations, albeit rare, are among the most life-threatening complications in BD. Pericarditis, coronary artery stenosis and/or aneurysm, myocarditis, cardiomyopathy, congestive heart failure, valvular pathology, endocarditis, intracardiac thrombosis, and aneurysm of aorta or its branches are major problems in this regard [119].

Estimated incidence of cardiac involvement reported 1%–5% in a case series. Mortality is rather high (around 20%). Cardiac involvement in BD could be asymptomatic [119].

Pericarditis is the most common cardiac manifestation in BD. Acute pericarditis, tamponade, constrictive pericarditis have been reported [119].

Coronary artery disease is rare in BD. It is more common in males younger than 40 years of age. CAD can lead to clinical manifestation of myocardial infarction, silent ischemia, and stable or unstable angina. Aneurysm, stenosis, and occlusion of arteritis are the most common etiologies for CAD in BD [119]. Coronary aneurysms are more frequent than the stenosis and can present as acute coronary syndrome and myocardial infarction, but sometimes are symptomatic [119].In young adults with myocardial infarction, BD should be considered as a nonatherosclerotic cause of CAD. Coronary arteritis may lead to myocardial infarction, but in some of patients with MI, coronary artery is normal. It seems that severe BD cases are to be more prone to AMI. It was shown that occlusion is developed as a result of thrombosis formation in CAD, consequently producing AMI [119].

A few cases with intracardiac thrombus which often precedes other manifestations of BD have been reported [119]. These thrombi are found mainly in the right ventricle and are often associated with pulmonary artery aneurysm [119]. It looks that endomyocardial fibrosis plays a role in the intracardiac thrombus development in some patients [119], Due to high specificity of right heart thrombus in BD, in any patient with this finding, diagnosis of BD should be considered [119]. Intracardiac thrombus is the major differential diagnosis when a young patient presents with an intracardiac mass [119]. This makes enormous diagnosis of cardiac myxoma in some instances [119]. It is especially common in young adults BD patients in the Middle East [119]. It is difficult to specify the mechanism of intracardiac thrombi formation. Good prognosis has been reported contrary to several recurrences of thrombosis [119]. Intracardiac thrombus can lead to superior vena cava syndrome [119] and pulmonary embolism [119].

Endocardial involvement may present with mitral and aortic valve prolapse, mitral or aortic insufficiency, aneurysm of sinus Valsalva, and endocarditis mimicking bacterial endocarditis [119]. The fibrosis secondary to endocardial involvement in BD may predispose to intracardiac thrombus formation [119]. It was reported that valvular prolapse including mitral valve prolapse can be related to vasculitis and tissue derangement [119]. Most of the aneurysm of the sinus Valsalva has been found in right coronary sinus which project into the right atrium or ventricle [119]. Usually the problem is diagnosed after rupture of aneurysm. A few cases of sinus Valsalva aneurysm in BD have been reported [119]. Usually they occurred in active phase of BD and are enlarging.Heart failure due to ruptured aneurysm requires urgent surgical repair [119].

Conductive abnormalities were also reported in several papers in the past [119] that they could directly be attributable or even nonrelated to BD per se [119].

## 15. Conclusion

In conclusion, cardiac manifestations of rheumatologic conditions/diseases can occur frequently in different conditions. They can be asymptomatic, or mild to severe. Rheumatologic diseases can involve the heart and vessels, as well as the musculoskeletal system. It is important to consider cardiac manifestations of rheumatologic conditions, as such, careful attention should be given to these conditions to prevent wrong diagnosis and treatment.

## Figures and Tables

**Figure 1:**
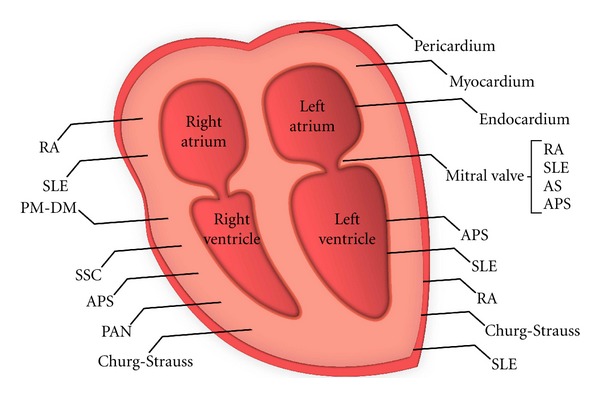
Specific rheumatic diseases that are associated with heart involvement.
